# Immunomodulatory Potential of Human Adipose Mesenchymal Stem Cells Derived Exosomes on *in vitro* Stimulated T Cells

**DOI:** 10.3389/fimmu.2014.00556

**Published:** 2014-11-04

**Authors:** Rebeca Blazquez, Francisco Miguel Sanchez-Margallo, Olga de la Rosa, Wilfried Dalemans, Verónica Álvarez, Raquel Tarazona, Javier G. Casado

**Affiliations:** ^1^Stem Cell Therapy Unit, Minimally Invasive Surgery Centre Jesus Uson, Cáceres, Spain; ^2^Research and Development Department, TiGenix SA, Parque Tecnológico de Madrid, Madrid, Spain; ^3^Technical Operations, TiGenix NV, Leuven, Belgium; ^4^Immunology Unit, Department of Physiology, University of Extremadura, Cáceres, Spain

**Keywords:** exosomes, mesenchymal stem cells, immunomodulation, lymphocytes, lymphocyte activation

## Abstract

In the recent years, it has been demonstrated that the biological activity of mesenchymal stem cells (MSCs) is mediated through the release of paracrine factors. Many of these factors are released into exosomes, which are small membranous vesicles that participate in cell–cell communication. Exosomes from MSCs are thought to have similar functions to MSCs such as repairing and regeneration of damaged tissue, but little is known about the immunomodulatory effect of these vesicles. Based on an extensive bibliography where the immunomodulatory capacity of MSCs has been demonstrated, here we hypothesized that released exosomes from MSCs may have an immunomodulatory role on the differentiation, activation and function of different lymphocyte subsets. According to this hypothesis, *in vitro* experiments were performed to characterize the immunomodulatory effect of human adipose MSCs derived exosomes (exo-hASCs) on *in vitro* stimulated T cells. The phenotypic characterization of cytotoxic and helper T cells (activation and differentiation markers) together with functional assays (proliferation and IFN-γ production) demonstrated that exo-hASCs exerted an inhibitory effect in the differentiation and activation of T cells as well as a reduced T cell proliferation and IFN-γ release on *in vitro* stimulated cells. In summary, here we demonstrate that MSCs-derived exosomes are a cell-derived product that could be considered as a therapeutic agent for the treatment of inflammation-related diseases.

## Introduction

Exosomes are small membranous vesicles secreted by most cell types. These vesicles participate in cell–cell communication and their content consists of RNA, lipids, and proteins. Some of these proteins (i.e., CD9, CD63, or CD81) are ubiquitously expressed, but depending on the cell source, cell type-specific proteins can be found being responsible of their functionality. The proteins, lipids, and RNA expression of exosomes from different cells and organisms are extensively described in ExoCarta database (1).

Exosomes can be easily isolated by ultracentrifugation from *in vitro* cultured cells but different isolation protocols have been described in the literature (2). All these protocols differ from each other on the basis of particular types of research being divided as procedures for discovery, diagnostic, or preparative research (3). For a clinical-grade production of exosomes, safe technologies for large scale production are an absolute prerequisite (4).

In preclinical settings, especially in murine models, exosomes have been applied for the treatment of many different diseases such as infections (5, 6), allergies (7) as well as autoimmune diseases (8, 9). Regarding the immunomodulatory potential of these vesicles, the first *in vivo* studies were conducted by Pêche et al. using bone marrow dendritic cell-derived exosomes (10, 11). Compared to preclinical studies, only a few clinical trials have been conducted using exosomes. Some of the first clinical trials were conducted in cancer patients using dendritic cell-derived exosomes (12) and ascites-derived exosomes (13) where the safety, tolerability, and efficacy of the treatments were demonstrated.

At the present, the therapeutic potential of exosomes derived from MSCs (Exo-MSCs) has been successfully applied in murine models for the treatment of cardiovascular diseases (14). In this sense, the proangiogenic effect described in different stem cell subsets may be the responsible of this therapeutic effect (15).

There are no differences in terms of morphological features, isolation, and storage conditions between exosomes derived from MSCs and other sources. As to the identification, exo-MSCs express not only the common surface markers of exosomes, such as CD9 and CD81, but also some adhesion molecules, including CD29, CD44, and CD73, which are expressed on the membrane of MSCs (16).

Accumulative evidences have established that, the effect of MSC transplantation is thought to be mediated in part, by a paracrine effect. Indeed, in the context of myocardial infarct it was experimentally quantified that the overall beneficial effect of paracrine mechanisms accounted between 50 and 80% (17). Several advantages of using released factors from MSCs have been described. For example, transferred cells may die or not fully home into the site of damaged tissue whereas biological factors can be locally administered with a controlled dosage (18).

Current preclinical trials with exo-MSCs have been driven for repairing damaged tissues, but few reports have been focused on the immunomodulatory effect of these vesicles. Here, we hypothesize that exo-MSCs may have similar regulatory functions than the original MSCs source on the differentiation, activation and function of different T cell subsets (16).

Supporting this idea, previous reports have demonstrated that the immunomodulatory capacity of MSCs against NK cells (19, 20), cytotoxic T lymphocytes (21), γδ T cells (22), dendritic cells (23, 24), or invariant NKT cells (25) is mediated by a paracrine mechanism.

In order to address this hypothesis, *in vitro* experiments were performed to characterize the immunomodulatory effect of exo-MSCs on *in vitro* stimulated T cells. The phenotypic characterization of cytotoxic and helper T cells (activation and differentiation markers) together with functional assays (proliferation and IFN-γ production) demonstrated that exo-MSCs exerted an inhibitory effect in the differentiation and activation of T cells as well as a reduced proliferation and IFN-γ release on *in vitro* expanded T cells. In summary, our results suggest that, exo-MSCs are a cell-derived product that could be considered as an immunomodulatory therapeutic agent for the treatment of immunological diseases.

## Materials and Methods

### Human adipose mesenchymal stem cells isolation and expansion

The human adipose mesenchymal stem cells (hASCs) were isolated from lipoaspirates obtained from human adipose tissue from healthy adult donors. Lipoaspirates were washed with PBS, and digested with collagenase type I in PBS. The digested sample was washed with 10% of fetal bovine serum (FBS), treated with ammonium chloride 160 mM, suspended in culture medium (DMEM containing 10% FBS), and filtered through a 40 μm nylon mesh. Cells were seeded onto tissue culture flasks and expanded at 37°C and 5% CO_2_, changing the culture medium every 7 days. Cells were passed to a new culture flask when cultures reached 90% of confluence. In addition, hASCs were tested by flow cytometry using specific surface markers being negative for CD14, CD31, CD34, CD45 and positive for CD29, CD59, CD90, and CD105 (data not shown). Cell lines from two healthy donors were used in the study. The biological samples were obtained after informed consent under the auspices of the appropriate Research and Ethics Committees.

### Isolation and purification of exosomes from hASCs

An enriched fraction of exosomes from hASCs (exo-hASCs) was obtained from hASCs cultured in 175 cm^2^ flasks. When cells reached a confluence of 80%, culture medium (DMEM containing 10% FBS) was replaced by exosome isolation medium (DMEM containing 1% insulin–transferrin–selenium). The hASCs supernatants were collected every 3–4 days. Exosomes were isolated from supernatants by two successive centrifugations at 1000 × *g* (10 min) and 5000 × *g* (20 min) at 4°C to eliminate cells and debris, followed by an ultracentrifugation at 100,000 × g for 6 h to precipitate exosomes. The pellets were resuspended in 250 μL of PBS and stored at −20°C. Prior to *in vitro* experiments, exosomes were quantified by Bradford assays and characterized by nanoparticle tracking analysis.

### Characterization of exo-hASCs

The concentration and size of purified exosomes were measured by nanoparticle tracking analysis (NanoSight Ltd, Amesbury, UK) that relates the rate of Brownian motion to particle size. Results were analyzed using the nanoparticle tracking analysis software package version 2.2. Triplicate samples were diluted 1:10 in sterile-filtered PBS and analyzed.

### Bradford assay

Exosome concentrations were indirectly measured by protein quantification in a Bradford assay. To quantify protein concentration, 20 μL of exosomes sample were incubated with 180 μL of Bradford reagent (Bio Rad Laboratories, Hercules, CA) at RT. Absorbance was read 5 min after at 595 nm, and protein concentration was extrapolated from a standard concentration curve of Bovine Serum Albumin.

### Lymphocytes isolation and preservation

Peripheral blood lymphocytes (PBLs) from healthy donors were obtained by centrifugation over Histopaque-1077 (Sigma, St. Louis, MO, USA) and washed twice with PBS. The PBLs were frozen and stored in liquid nitrogen. For *in vitro* experiments, cell aliquots were thawed at 37°C, added to 10 mL of RPMI 1640 and centrifuged at 1500 rpm for 5 min to eliminate DMSO. Pellet was resuspended in RPMI 1640 supplemented with 10% of FBS.

### *In vitro* stimulation of T cells and co-culture with exosomes

To determine the immunomodulatory effect of exo-hASCs on *in vitro* stimulated PBLs, 2 × 10^5^ purified PBLs were seeded in a 96 wells plate (200 μl per well). To stimulate PBLs, a T cell activation/expansion kit (Miltenyi Biotec Inc, San Diego, CA, USA) was used, adding 5 μL of microbeads coated with anti-CD2/anti-CD3/anti-CD28 to each well. Finally, exosomes at different concentrations (4, 8, and 16 μg/10^6^ PBLs) were added to wells. The PBLs were cultured for 6 days. Negative controls (non-stimulated PBLs) and positive controls (stimulated PBLs without exosomes) were used in all the experiments.

### CFSE proliferation assay

The proliferative behavior of T cells was quantified by carboxyfluorescein succinimidyl ester (CFSE) dilution. The CFSE staining was performed before seeding, using the CFSE cell proliferation kit (Invitrogen, Eugene, OR) at a final concentration of 10 μM for 10 min at 37°C, followed by immediate quenching with culture medium. After 6 days, *in vitro* stimulated PBLs in the presence or absence of exo-hASCs were tested for CFSE dilution by flow cytometry.

### Differentiation/activation markers expression analysis on *in vitro* stimulated PBLs

For flow cytometric analysis of *in vitro* stimulated PBLs, the cells were collected from wells after 6 days by pipetting up and down. The cells were stained with fluorescence-labeled human monoclonal antibodies against CD3 (SK7), CD4 (SK3), CD8 (SK1), CCR7 (3D12), CD45RA (L48) (BD Biosciences, San Jose, CA, USA). The markers expression analysis was performed as follows: 2 × 10^5^ cells were incubated for 30 min at 4°C with appropriate concentrations of monoclonal antibodies in the presence of PBS containing 2% FBS. The cells were washed and resuspended in PBS. The flow cytometric analysis was performed on a FACScalibur cytometer (BD Biosciences, San Jose, CA, USA) after acquisition of 10^5^ events. Cells were primarily selected using forward and side scatter characteristics and fluorescence was analyzed using CellQuest software (BD Biosciences, San Jose, CA, USA). Isotype-matched negative control antibodies were used in all the experiments. The mean relative fluorescence intensity was calculated by dividing the mean fluorescent intensity (MFI) by the MFI of its negative control.

### Intracellular gamma-interferon assay

For IFN-γ assays, the PBLs were *in vitro* stimulated with the T cell activation/expansion kit (Miltenyi Biotec Inc, San Diego, CA, USA) for 6 days in the presence of exo-hASCs at 16 μg/10^6^ PBLs. The PBLs were then incubated for 6 h with BD GolgiStop. PBLs were stained with PerCP-labeled anti-CD4 (SK3) and APC-labeled anti-CD8 (SK1), fixed and permeabilized using BD Cytofix/Cytoperm fixation/permeabilization kit. Finally, cells were stained with PE-labeled anti-IFN-γ antibody (all reagents from BD Biosciences, San Jose, CA, USA). Analysis by flow cytometry was performed by measuring the frequency of IFN-γ expression on gated CD3^+^CD4^+^ and CD3^+^CD8^+^ cells.

### Statistical analysis

Data were statistically analyzed using the Student’s *t*-test for variables with parametric distribution. For the proliferation assay, an ANOVA with *post hoc* Bonferroni test was performed. The *p*-values ≤0.10 or ≤0.05 were considered statistically significant. All the statistical determinations were made using SPSS-21 software (SPSS, Chicago, IL, USA).

## Results

### Size distribution and concentration of exo-hASCs

**Figure 1:**
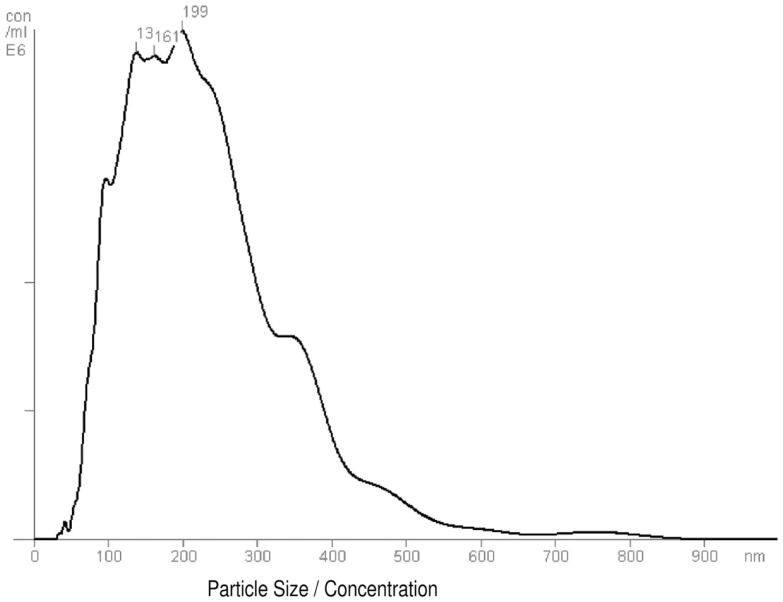
**Frequency size distribution graph of exo-hASCs**. The nanoparticle tracking analysis was performed on exosome samples to quantify size distribution and particle concentration (*n* = 6). A representative graph of nanoparticle tracking analysis is shown.

An enriched fraction of exosomes was collected from hASCs by ultracentrifugation. The protein concentration of exosomes was determined by Bradford assay. Three independently performed nanoparticle tracking analysis were performed for each exosome sample to quantify size distribution and particle concentration. Firstly, the total protein concentration allowed us to quantify exosomes for *in vitro* assays. Secondly, the nanoparticle tracking analysis allowed us to characterize the released vesicles. The size of isolated vesicles ranged from 223 to 282 nm and the mean size and standard deviation was 246.8 ± 25.05 nm. Representative results of exo-hASCs are displayed as a frequency size distribution graph (Figure 1). The corresponding nanoparticle tracking analysis video frame is included as Video S1 in Supplementary Material. Finally, the concentration of exosomes (*n* = 6) was determined by nanoparticle tracking analysis and ranged between 8.4 and 9.7 (×10^9^) particles per milliliter and the mean concentration was 9.1 ± 0.5 (×10^9^) particles per milliliter.

### Proliferative ability of *in vitro* stimulated T cells co-cultured in the presence of exo-hASCs

**Figure 2:**
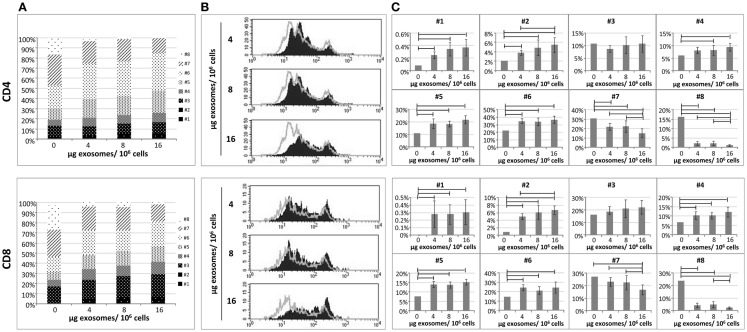
**The proliferative ability of *in vitro* stimulated PBLs is reduced by exo-hASCs**. The PBLs were cultured either alone or co-cultured with different batches of exo-hASCs (*n* = 8) at different concentrations (4, 8, and 16 μg of exosomes per million of PBLs). At day six, PBLs were collected and T-lymphocytes subsets were stained with anti-CD3, anti-CD4, and anti-CD8. Fluorescence profiles of CFSE-labeled cells allowed us to identify eight divisions. A detailed representation of CD4^+^ T cells and CD8^+^ T cells showing the percentage of the total population in each cell division cycle (indicated as #) is provided **(A)**, as well as a representative histogram **(B)**. The statistical comparison of lymphocyte subsets at different cell division cycles is also provided **(C)**. Horizontal bars represent statistically significant differences between the groups (significant at *p* ≤ 0.05).

In order to assess the biological activity of exo-hASCs, we aimed to determine their effect over the proliferation rate of lymphocyte subsets. For that, a total of 2 × 10^6^ PBLs were stimulated with anti-CD2/anti-CD3/anti-CD28 as described in Section “Materials and Methods” and co-cultured with different concentrations of exo-hASCs (4, 8, and 16 μg/10^6^ PBLs) during 6 days. The proliferation ability was determined by CFSE dilution. Non-stimulated PBLs were used as negative control, and stimulated PBLs without exosomes constituted the positive control. As expected, the proliferation rate of non-stimulated PBLs was very low (data not shown) and the maximum proliferation rate was reached by stimulated PBLs without exosomes. A total of eight cell divisions were detected by CFSE fluorescence. As shown in the Figure 2A, when *in vitro* stimulated lymphocytes were cultured in the presence of different concentrations of exo-hASCs, the proliferation rate was proportionally decreased both in CD4^+^ and CD8^+^ T cells. A large percentage of cells presented a low number of cell divisions, while the highest number of cell divisions was reached by a lower percentage of cells. A detailed representation showing the percentage of cells in each division cycle is provided in the Figure 2A. A representative histogram (Figure 2B) and a detailed representation showing the percentage of cells in each division cycle is also provided (Figure 2C).

Here, it can be seen how increasing concentrations of exosomes are arresting both CD4 and CD8 proliferation from eight generations to seven. Moreover exosomes are retaining the cells in the earlier division cycles 4, 5, and 6, in where the percentage of cells are significantly higher in the presence of exosomes, however, division cycles 7 and 8 have a significantly reduced percentage of cells when higher doses of exosomes were used. The first two division cycles contain a very low percentage of T cells both in the presence or absence of exosomes indicating that the effect of the polyclonal stimulation starts after these two division cycles, nevertheless the presence of exosomes are still significantly retaining cells in these firs two division cycles (although this is happening in a group of T cells below 10%). The statistical analysis showed that, significant differences were found in different division cycles either in CD4^+^ and CD8^+^ T cells. Finally, the stimulation index was calculated on CD4^+^ and CD8^+^ T cells as frequencies of CFSE-low T cells among unstimulated T cells. The stimulation index of CD4^+^ and CD8^+^ T cells stimulated with anti-CD2/anti-CD3/anti-CD28 was 692.3 and 655.6, respectively. However, when PBLs were stimulated in the presence of exosomes, the stain index significantly decreased on CD4^+^ T cells (589.93 ± 39.31, 585 ± 80.27, 529.14 ± 58.88 at 4, 8, and 16 μg) as well as in CD8^+^ T cells (519.75 ± 60.97, 488.03 ± 107.32, 437.4 ± 79.25 at 4, 8, and 16 μg).

### T cells subsets distribution of *in vitro* stimulated T cells co-cultured in the presence of exo-hASCs

**Figure 3:**
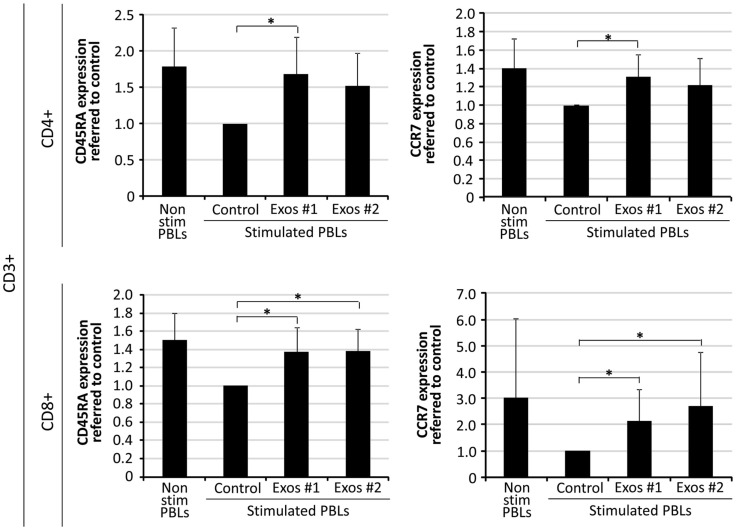
**Percentage of CD45RA and CCR7 expression on *in vitro* stimulated T cells co-cultured in the presence of exo-hASCs**. At day 6, *in vitro* stimulated PBLs were analyzed for CD45RA and CCR7 on CD8^+^ and CD4^+^ T cell subsets. Two different exo-hASCs at 16 μg/10^6^ cells from different donors were used in these experiments (Exos#1 and Exos#2). The graphs show the normalized quantitative expression referred to control (*in vitro* stimulated T cells in the absence of exo-hASCs). Values shown in the bars represent mean ± SD of three independently performed experiments. Horizontal bars represent statistically significant differences between the stimulated PBLs groups (significant at *p* ≤ 0.1).

The CD45RA isoform and chemokine receptor CCR7 are surface marker commonly used to identify the differentiation stages of CD4^+^ and CD8^+^ T cells. In order to study the effect of exo-hASCs over lymphocyte subsets, a total of 2 × 10^6^ stimulated PBLs were cultured in the presence of exo-hASCs (from two different donors) at 16 μg/10^6^ PBLs. At day 6, flow cytometry was performed using a commercial antibody against CD45RA and CCR7. The quantitative expression of CD45RA and CCR7 was normalized referred to control (*in vitro* stimulated T cells in the absence of exo-hASCs). Our results showed a significant decrease CD45RA^+^ and CCR7^+^ cells both in the CD4^+^ and CD8^+^ T cells in the positive control (stimulated PBLs). However, the loss of CD45RA and CCR7 on *in vitro* stimulated PBLs was partially compensated by the presence of exo-hASCs (Figure 3). Representative histograms are provided in Figure S1 in Supplementary Material.

**Figure 4:**
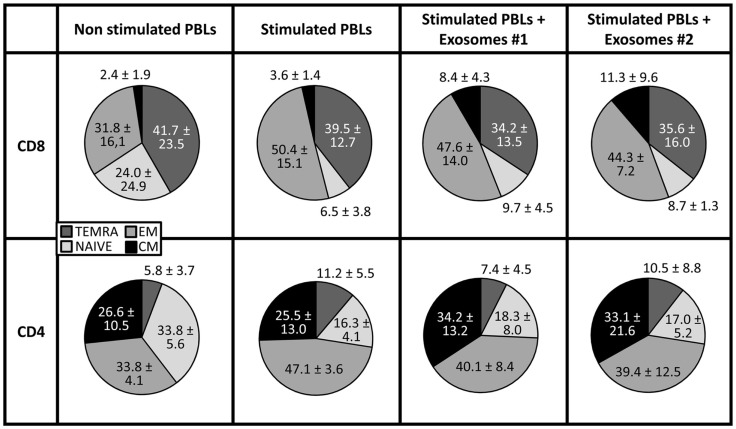
**CD45RA and CCR7 co-expression on *in vitro* stimulated T cells co-cultured in the presence of exo-hASCs**. At day 6, *in vitro* stimulated PBLs were analyzed for the co-expression of CD45RA and CCR7. The CD45RA isoform and CCR7 distinguishes four subsets of T cells: terminally differentiated RA^+^ T cells (TEMRA, CD45RA^+^ CCR7^−^), naïve T cells (NAIVE, CCR7^+^ CD45RA^+^), and two memory subsets: effector memory (EM, CD45RA^−^ CCR7^−^) and central memory (CM, CD45RA^−^ CCR7^+^). Two different exosomes from different donors were used in these experiments (Exos#1 and Exos#2). Values shown represent mean ± SD of 3 independently performed experiments.

In the model proposed by Lanzavecchia and Sallusto, four different stages have been defined within CD8^+^ T cells according to the combined analysis of CD45RA and CCR7 expression, namely: naïve (CD45RA^+^CCR7^+^), central memory (CD45RA^−^CCR7^+^) and at least two subset of effector-memory cells: effector-memory cells (CD45RA^−^CCR7^−^) and terminally differentiated effector-memory cells (CD45RA^+^CCR7^−^) (26, 27). To study the effect of exo-hASCs over this distribution, the co-expression of CD45RA and CCR7 was analyzed by flow cytometry on CD4^+^ and CD8^+^ T cell subsets. As shown in Figure 4, although the percentage of naïve cells was not significantly modified by the presence of exo-hASCs, a significant decrease of terminally differentiated effector-memory cells (CD45RA^+^CCR7^−^) was observed on *in vitro* stimulated CD8^+^ T cells cultured in the presence of exo-hASCs. In the case of CD4^+^ T cells, exo-hASCs reduced the percentage of effector-memory cells (CD45RA^−^CCR7^−^) and significantly increased the percentage of central memory cells (CD45RA^−^CCR7^+^).

### IFN-γ production on *in vitro* stimulated T cells co-cultured in the presence of human adipose mesenchymal stem cells derived exosomes

**Figure 5:**
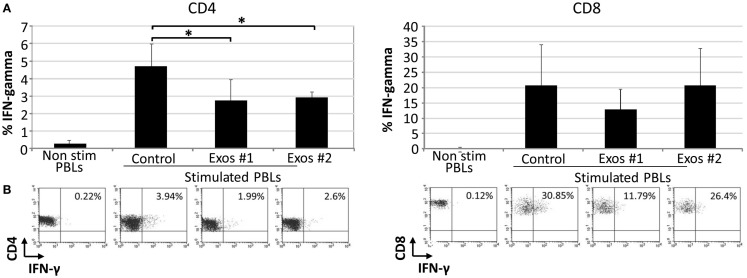
**The exo-hASCs inhibit the IFN-γ production of *in vitro* stimulated T cells**. Two different exosomes from different donors were used in these experiments (Exos#1 and Exos#2). Graphs represent the mean ± SD of 3 independently performed experiments. A representative dot plot of each condition is represented below each graph, and numbers in the quadrants indicate the percentage of IFN-γ in gated CD4^+^ **(A)** and CD8^+^ T cells **(B)** (significant at *p* ≤ 0.05).

The IFN-γ is a pro-inflammatory cytokine secreted by immune cells under certain conditions of activation. There is a direct correlation between IFN-γ secretion and the level of T cell activation. In order to determine the effect of exosomes on the secretory IFN-γ response of T cells, PBLs were cultured in the presence and absence of exo-hASCs during 6 days and intracellular levels of IFN-γ were determined on CD4^+^ and CD8^+^ T cell subsets. Our results showed that, at day 6, the percentage of intracellular IFN-γ was reduced when PBLs were cultured with exosomes, in comparison to positive control, in both T cell subsets. However, this reduction was only statistically significant on gated CD4^+^ T cells (Figure 5). These results demonstrated that exo-hASCs impaired not only the differentiation phenotype of lymphocytes but also their IFN-γ secretion.

## Discussion

The hASCs have been successfully used for the treatment of numerous diseases (28). These cells can be isolated from the adult liposuctioned tissue and efficiently expanded *in vitro* to be used as an “off-the-shelf” cellular product. At the present, there are quite a few stem cell products in the market (29) but hASCs are currently considered one of the most promising cell types for therapeutic applications that fulfill regulatory requirements.

The hASCs have immunomodulatory properties, which are directly mediated by cell–cell contact or indirectly mediated through the release of immunosuppressive factors. The release of these factors is enhanced under inflammatory conditions and results in suppression of T cell function (proliferation, differentiation, and cytotoxicity) (30), B cell functions (31), NK cell cytotoxicity (20), decrease in maturation, and activation of dendritic cells (32) as well as an increase of regulatory T cells (33, 34).

Among the released factors, the exosomes from MSCs have been considered as a promising candidate for a novel cell-free therapy. At the present, these microvesicles have been tested in preclinical settings for the treatment of cardiovascular diseases (35), kidney injury (36), graft-versus-host disease (37), and neurological diseases (38). In clinical settings, the exo-MSCs have been tested in graft-versus-host disease patients, which experienced improvement in symptoms for 5 months (39).

In these studies, exosomes from MSCs have demonstrated a biological effect for repairing tissue damage. From an immunological point of view, these exosomes have demonstrated immunomodulatory properties inducing peripheral tolerance (40) and modulating the immune response (41).

In this work, we aimed to investigate the immunomodulatory role of an enriched fraction of exo-hASCs on T cell subsets under *in vitro* conditions. For this purpose, the T cells were *in vitro* expanded and activated with anti-CD2, anti-CD3, and anti-CD28 that partially mimic stimulation by antigen-presenting cells (42). The immunomodulation was assessed by measuring the proliferative behavior of T cells, their differentiation toward the memory lineage and IFN-γ secretory response.

Although this is a preliminary study, our results demonstrated that exo-hASCs significantly inhibited the proliferation of CD4 and CD8 T cells. These results are very similar to our previously published results using hASCs co-cultured with *in vitro* activated PBLs (30). Recent reports have demonstrated that TNF-α/NF-κB signaling in MSCs are required for the inhibition of T-cell proliferation (43). In this sense, future studies will be conducted to evaluate if the anti-proliferative activity of exo-hASCs could also be related with the activation of NF-κB.

Together with the inhibition of T cell proliferation, here we hypothesized that exo-hASCs may arrest the T cell differentiation toward effector or memory cell phenotypes. In order to confirm this hypothesis, *in vitro* activated lymphocytes were co-cultured in the presence of exo-hASCs and the differentiation profile of CD4^+^ and CD8^+^ T cells was monitored according to co-expression of CD45RA and CCR7 molecules. The combined usage of CD45RA and CCR7 allowed us the identification of different CD8^+^ T cell subsets (26) and CD4^+^ T cell subsets (27): naive (CCR7^+^CD45RA^+^), central memory (CCR7^+^CD45RA^−^), effector memory (CCR7^−^CD45RA^−^), and terminally differentiated effector-memory cells (CD45RA^+^CCR7^−^). Our results evidenced that exo-hASCs hamper the *in vitro* differentiation mediated by anti-CD3/CD2/CD28 stimuli. Actually, in the case of CD8^+^ and CD4^+^ T cells, exo-hASCs have an inhibitory effect in the differentiation of toward a terminally differentiated phenotype and effector-memory phenotype, respectively.

These *in vitro* results with exo-hASCs showed similarities with previous reports using MSCs and *in vitro* stimulated PBLs where the T lymphocytes showed reduced-memory responses after a tetanus toxoid boost (44). Current experiments are being conducted in a relevant animal model of T cell-mediated disease (collagen-induced arthritis experimental model for rheumatoid arthritis). Once completed, these results will give us a more complete understanding of exo-hASCs as a therapeutic agent for the control of local T cell responses.

Finally, the immunomodulatory activity of exo-hASCs was determined by measuring IFN-γ production on CD4^+^ and CD8^+^ T cells. These experiments confirmed that, similarly to *in vitro* experiments using hASCs, both in contact or separated by transwells (30), the IFN-γ production was significantly reduced by exo-hASCs. Considering that IFN-γ is crucial for protection against immune-mediated inflammatory disorders, we could assume that exo-hASCs could be used as ideal vehicles for a local immunosuppression. Moreover, in contrast to cell therapy, where the viability, homing, or implantation of individual cells is compromised, the usage of well-characterized exo-hASCs in a dosing regimen that can be controlled and defined in space and time could be considered an advantage (45). Additionally, several authors have reported the susceptibility of allogeneic cells to CD8^+^ T cells and NK cells, which is an important issue for the clinical efficacy of MSCs (46). In the case of exo-hASCs, these microvesicles will not be affected by cell-mediated lysis, which is an advantage for their therapeutic effectiveness.

An important aspect to be discussed here is the role of MHC molecules on exosomes. It has been previously described by Pêche et al. that, exosomes from bone marrow dendritic cells (exo-DCs) induce regulatory responses and allograft tolerance through the presentation of donor-MHC antigens (10). These exo-DCs are positives for MHC class I, MHC class II, and co-stimulatory molecules and the *in vivo* administration of allogeneic exosomes induced tolerance (11).

Contrary to exo-DCs, the exo-hASCs are characterized by the absence of MHC class II and co-stimulatory molecules. Regarding to MHC class I, the hASCs cell lines contains very low levels of MHC class I and the expression of this molecule in exo-hASCs is still under debate. Indeed, in Exocarta (a database of exosomal proteins, http://www.exocarta.org/), the presence of HLA-A, HLA-B, or HLA-C has not been detected in the 939 proteins analyzed from exo-MSCs. However, the proteomic analysis of exosomes from human embryonic stem cell-derived MSCs have demonstrated the presence of HLA-A molecules in these vesicles (47).

On the other hand, considering that human clinical trials using allogeneic hASC is not causing early, aggressive immunological rejection, we could assume that allogeneic exo-hASCs from non-activated hASCs might have similar consequences. The absence of MHC class II and co-stimulatory molecules on exo-hASCs may also indicate that these vesicles have a direct inhibitory effect on T cells being independent from antigen presentation. Nevertheless, although the inhibitory effect was only tested in allogeneic setting (using exo-hASCs from clinically established hASCs), future experiments are being conducted to determine if inhibitory effect is stronger in autologous T cells.

The limitations of the study are evident as this paper has been purely focused on the *in vitro* role of exo-hASCs against *in vitro* activated T cell subsets, and future experiments should confirm these observations on *in vivo* animal models. These *in vivo* animal models will help us to define the optimal the route, dosing and frequencies of exo-hASCs administration.

In summary, this paper provides a better understanding of exo-hASCs for their future applicability in clinical practice. Moreover, in terms of immunomodulation, our *in vitro* results demonstrated a parallelism between hASCs and exo-hASCs.

## Conflict of Interest Statement

Olga de la Rosa and Wilfried Dalemans are full time employees of TiGenix. The other co-authors declare that the research was conducted in the absence of any commercial or financial relationships that could be construed as a potential conflict of interest.

## Supplementary Material

The Supplementary Material for this article can be found online at http://www.frontiersin.org/Journal/10.3389/fimmu.2014.00556/abstract

Supplementary Video S1 **Nanoparticle tracking analysis video frame**. A representative video of nanoparticle tracking analysis of exo-hASCs is shown.

Supplementary Figure S1 **Percentage of CD45RA and CCR7 expression on in vitro stimulated T cells co-cultured in the presence of exo-hASCs**. At day 6, *in vitro* stimulated PBLs were analyzed for CD45RA and CCR7 on CD8^+^ and CD4^+^ T cell subsets. Two different exo-hASCs at 16 μg/10^6^ cells from different donors were used in these experiments (Exos#1 and Exos#2). The figure shows representative histograms from the three different experiments. Percentage of positive cells is shown in each histogram.
