# Supplementary figures and images for: Immunomodulatory Potential of Human Adipose Mesenchymal Stem Cells Derived Exosomes on in vitro Stimulated T Cells
Source: Front Immunol. 2014 Nov 4;5:556. doi: 10.3389/fimmu.2014.00556 (PMC4220146; doi:10.3389/fimmu.2014.00556)

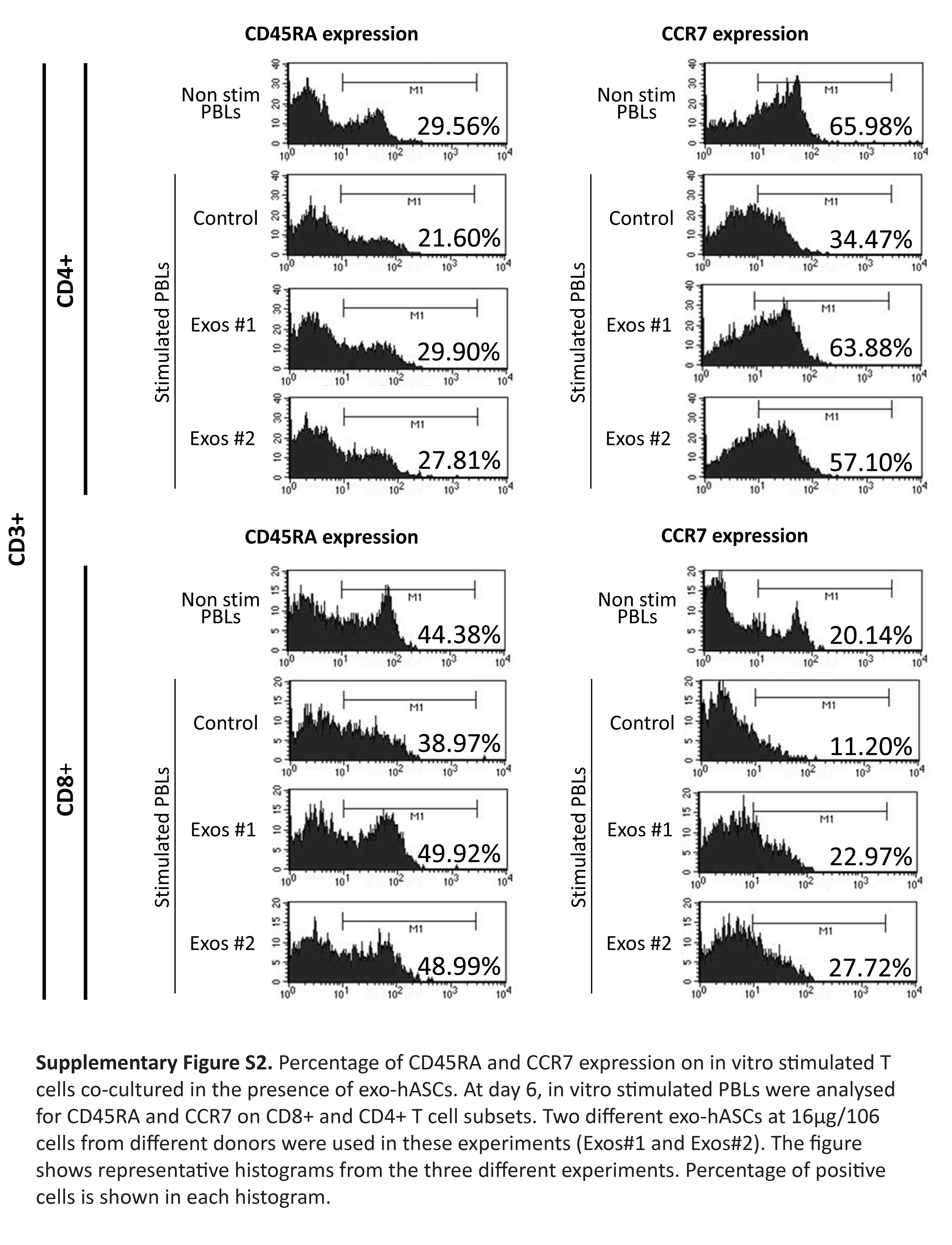

Supplement: Supplementary Figure S1 — Percentage of CD45RA and CCR7 expression on in vitro stimulated T cells co-cultured in the presence of exo-hASCs. At day 6, in vitro stimulated PBLs were analyzed for CD45RA and CCR7 on CD8+ and CD4+ T cell subsets. Two different exo-hASCs at 16 μg/106 cells from different donors were used in these experiments (Exos#1 and Exos#2). The figure shows representative histograms from the three different experiments. Percentage of positive cells is shown in each histogram. [file Image_1.TIF]
